# Who benefits from increased service utilisation? Examining the distributional effects of payment for performance in Tanzania

**DOI:** 10.1186/s12939-018-0728-x

**Published:** 2018-01-29

**Authors:** Peter Binyaruka, Bjarne Robberstad, Gaute Torsvik, Josephine Borghi

**Affiliations:** 1 Centre for International Health, University of Bergen, PO Box 7804, N-5020 Bergen, Norway; 2 Ifakara Health Institute, PO Box 78373, Dar es Salaam, Tanzania; 3 Chr. Michelsen Institute, PO Box 6033, Bergen, Norway; 4 Department of Economics, University of Oslo, PO Box 1095, Oslo, Norway; 5 Department of Global Health and Development, London School of Hygiene and Tropical Medicine, 15-17 Tavistock Place, London, WC1H 9SH UK

**Keywords:** Inequality, Equity, Social determinants of health, Universal coverage, Distributional effects, Healthcare financing, Pay for performance, Tanzania

## Abstract

**Background:**

Payment for performance (P4P) strategies, which provide financial incentives to health workers and/or facilities for reaching pre-defined performance targets, can improve healthcare utilisation and quality. P4P may also reduce inequalities in healthcare use and access by enhancing universal access to care, for example, through reducing the financial barriers to accessing care. However, P4P may also enhance inequalities in healthcare if providers cherry-pick the easier-to-reach patients to meet their performance targets. In this study, we examine the heterogeneity of P4P effects on service utilisation across population subgroups and its implications for inequalities in Tanzania.

**Methods:**

We used household data from an evaluation of a P4P programme in Tanzania. We surveyed about 3000 households with women who delivered in the last 12 months prior to the interview from seven intervention and four comparison districts in January 2012 and a similar number of households in 13 months later. The household data were used to generate the population subgroups and to measure the incentivised service utilisation outcomes. We focused on two outcomes that improved significantly under the P4P, i.e. institutional delivery rate and the uptake of antimalarials for pregnant women. We used a difference-in-differences linear regression model to estimate the effect of P4P on utilisation outcomes across the different population subgroups.

**Results:**

P4P led to a significant increase in the rate of institutional deliveries among women in poorest and in middle wealth status households, but not among women in least poor households. However, the differential effect was marginally greater among women in the middle wealth households compared to women in the least poor households (*p* = 0.094). The effect of P4P on institutional deliveries was also significantly higher among women in rural districts compared to women in urban districts (*p* = 0.028 for differential effect), and among uninsured women than insured women (*p* = 0.001 for differential effect). The effect of P4P on the uptake of antimalarials was equally distributed across population subgroups.

**Conclusion:**

P4P can enhance equitable healthcare access and use especially when the demand-side barriers to access care such as user fees associated with drug purchase due to stock-outs have been reduced.

## Introduction

Payment for performance (P4P) is a supply-side financing strategy which involves financial incentives being paid to health workers and/or facilities for reaching pre-defined performance targets. This approach started in high-income countries (HICs) with the aim of improving health care quality [24, 64, 65]. P4P is also increasingly being used in low- and middle-income countries (LMICs) to improve quality and use of health services, as well as to strengthen health systems [31, 57, 89]. The evidence base on the effectiveness of P4P is growing and suggests mixed effects with notable improvements for some incentivised indicators [9, 11, 17, 24, 26, 35, 61, 69, 73, 77].

However, most evaluations focus on average effects and pay little attention to distributional effects across provider or population subgroups [51]. There is, however, a growing awareness that average effects may mask important heterogeneous programme effects [12, 13, 19, 22, 38, 41, 51]. This study examines the heterogeneity of P4P effects on service utilisation across population subgroups. The overall goal is to display heterogeneous treatment effects, and specifically to check if the effects on population subgroups will reduce or enhance exiting inequalities in access to and utilisation of health care services.

Inequalities in access to and use of health services in favour of wealthier populations are still prevalent in many settings, with the greatest inequalities in the poorest settings [8, 15, 52, 56, 60, 68, 78, 79, 82, 84]. Factors referred to as “social determinants of health” such as economic status, education, location and age [21, 54, 60, 87], mostly drive these inequalities. From a theoretical point of view, it is hard to know how P4P will affect pre-existing inequalities. However, P4P can reduce inequalities in access to healthcare, for example, by encouraging providers to extend services to underserved groups (e.g. by reducing financial barriers to access care) in a bid to meet performance targets [31, 57]. On the other hand, P4P could also enhance inequalities in access to healthcare if providers cherry-pick the easier-to-reach patients in order to meet their performance targets [40].

Studies in HICs have found differential effects of P4P on healthcare quality between socioeconomic groups in favour of wealthier populations (pro-rich) but this effect declined over time. These studies have not found any differential effect with respect to age, sex and ethnicity [2, 14, 24, 80]. Evidence from LMICs is more limited and varied across service types [63]. For example, the effect of P4P on institutional delivery rates was greater among wealthier groups (pro-rich) in most settings [17, 46, 77] but there was an indication that it was greater among poorer groups (pro-poor) in Tanzania[11]. The effect of P4P on institutional deliveries was greater among women with health insurance in Rwanda [46] or a maternity care voucher in Cambodia [77] than their counterparts. The effect of P4P on family planning coverage was greater among wealthier groups (pro-rich), in Rwanda [46], and the effect on immunisation coverage was greater among poorer groups (pro-poor), in Burundi [17]. However, studies based on Rwanda Demographic Health Survey (DHS) data reported no differential effect by socioeconomic groups on the use of maternal care [62] and on child curative care seeking [72].

To date, most studies on differential effects of P4P have disaggregated the effect of P4P across population economic status particularly in LMICs, with little attention to other social determinants (e.g. education, occupation, and age), which are also known to affect the use of health services [4, 60], including maternal health services [30, 32, 71]. The assessment of programme differential effects across various social determinants in a broad perspective is crucial to inform universal access policies [28, 53, 60], and may help to understand how different service users are affected by a programme such as P4P [63]. In this paper, we examine the differential effect of P4P on service utilisation in Tanzania across a variety of population subgroups by stratified analyses according to various social determinants.

This paper proceeds as follows. The next section presents the conceptual framework, followed by the description of the P4P programme in Tanzania. The other sections include the methods and analysis, followed by the results, discussion and conclusion.

### Conceptual framework

P4P programmes give providers incentives to change their behaviour to improve the quality of care in order to enhance utilisation and obtain financial rewards [66]. Based on this logic P4P can improve average service utilisation and the distribution of improved utilisation across population subgroups through the *supply-side response* (how providers respond to incentives) and the resulting *demand-side response* that triggers (how patients respond to supply side changes).

#### Supply-side response

To meet performance targets aimed at increasing the quantity of services provided, providers are likely to adopt strategies to attract more patients to facilities [31, 57]. One such strategy could be to make services more affordable [57], for example by reducing user fees, or by reducing drug stock-outs, avoiding patients having to procure drugs privately [10, 11]. Another strategy could be to improve responsiveness to service users, for example, by being kinder during service delivery [11]. However, providers might also attempt to cherry-pick patients or focus on easy-to-reach populations (i.e. underserved but easily reached) in order to meet the performance targets [25, 40], leaving the hard-to-reach (i.e. poorest with greatest need) underserved. In fact, providers may need to exert greater effort and time to serve the hard-to-reach [37]. The efficiency gains in that case can be reached but at the expenses of inequity [47].

#### Demand-side responses

According to Andersen’s behavioural model of healthcare utilisation [3, 4], the use of health services is a function of patient’s propensity to use services (predisposing factors), factors that facilitate or impede access and use (enabling factors), as well as perceived need for healthcare (need factors). These factors among others are also social determinants of health [21, 54, 74]. The interactions between a P4P programme (supply-side response) and social determinants (demand-side factors) may affect the use and distribution of health services. For example, reduced financial barriers to access care, resulting from provider response to incentives, may stimulate demand especially for poor and/or uninsured individuals, since they are more responsive to a change in healthcare costs consistent with demand theory [33, 49]. Demand for health services may also increase if the quality of care supplied is improved [1]; for example, through increased drug availability and better interpersonal care [10, 11]. Better-off populations (e.g. wealthier, educated, and urban residents) may also benefit more from quality improvements simply because they use services more than their counterpart populations [8, 15, 21, 32, 54, 68, 81].

Despite the potential interactions between the demand and supply-side response to P4P, the health care sector does not operate like a classic free market [6, 61]. For example, the demand-side response may be weak when some demand-side barriers to access care (e.g. cultural and information barriers) are unaffected by the supply-side response to incentives [27, 48, 61, 88].

### P4P in Tanzania

In 2011, the Ministry of Health and Social Welfare (MoHSW) in Tanzania with support from the Government of Norway introduced a P4P scheme as a pilot in Pwani region. The scheme aimed to improve maternal and child health (MCH) and inform the national P4P roll out. Pwani is one of 30 regions in the country and has seven districts with more than 209 health facilities. It has a population of just over a million [59]. All health facilities providing MCH services in the region were eligible to implement the P4P scheme. The P4P scheme involved a series of performance targets for facilities that were set in relation to the coverage of specific services (e.g. institutional delivery) or for care provided during a service (e.g. uptake of antimalarials during antenatal care) (Table 1), as described in more detail elsewhere [11, 18]. Performance was rewarded based on two methods of target setting: single and multiple thresholds targets. The strategies to reach performance targets were left to the discretion of the health workers at the individual facilities. District and regional managers were also eligible to receive performance payouts based on the performance of the facilities in their district or region.

**Table 1:** Service indicators and performance targets for facilities implementing P4P in Tanzania

P4P service indicators	Method	Baseline coverage (previous cycle)				
		0–20%	21–40%	41–70%	71 − 85%	85%+
Coverage indicators						
% of institutional deliveries	Percentage point increase	15%	10%	5%	5%	Maintain 85%+
% of mothers attending a facility within 7 days of delivery.	Percentage point increase	15%	10%	5%	5%	Maintain 85%+
% of women using long term contraceptives	Percentage point increase	20%	15%	10%	Maintain above 71%	Maintain 85%+
% children under 1 year received measles vaccine	Overall result	50%	65%	75%	80%+	Maintain 85%+
% children under 1 year received Penta 3	Overall result	50%	65%	75%	80%+	Maintain 85%+
% of complete partographs	Overall result	80%	80%	80%	80%+	Maintain above 80%
HMIS reports submitted to district managers on time and complete	Overall result	100%	100%	100%	100%	100%
Content of care indicators						
% ANC clients receiving two doses of IPT	Overall result	80%	80%	80%	80%+	Maintain above 80%
% HIV+ ANC clients on ART	Overall result	40%	60%	75%	75%+	Maintain 85%+
% of children receiving polio vaccine (OPV0) at birth	Overall result	60%	75%	80%	80%+	Maintain 85%+

The United Republic of Tanzania, Ministry of Health and Social Welfare. 2011. The Coast Region Pay for Performance (P4P) Pilot: Design Document

85% + = 85% or more; 80% + = 80% or more; *HMIS* Health Management Information System, *ANC* Antenatal care

The extent to which facilities were successful in achieving performance targets determined the level of bonus payout they would receive as part of the programme. Full payment was made if 100% of a given target was achieved, and 50% of payment was made for 75% < 100% achievement, while no payment was made for lower levels of performance. The maximum payout if all targets were fully attained was USD 820 per cycle for dispensaries; USD 3220 for health centres and USD 6790 for hospitals. The payouts were additional to the funding facilities receive to cover operational costs and salaries of health workers. Incentive payouts at the facility-level included bonuses to staff (equivalent to 10% of their monthly salary if all targets were fully attained) and funds that could be used for facility improvement or demand creation initiatives (10% of the total in hospitals and 25% in lower level facilities). District and regional managers received bonus payments of up to USD 3000 per cycle.

To determine whether performance targets were met, performance data were compiled by facilities and verified by the P4P implementing agency every six months (one cycle) before distributing payouts.

The P4P programme was the subject of a process and impact evaluation. The impact evaluation showed a significant positive effect on two out of eight incentivised service indicators: institutional delivery rate and provision of antimalarial during antenatal care [11]. P4P was also associated with a number of process changes such as increased availability of drugs and supplies, increased supportive supervision, a reduced chance of paying user fees, and greater provider kindness during delivery care [5, 10, 11, 55].

## Methods

### Study design

Our study used data from a controlled before and after evaluation study of the P4P scheme in Pwani region, Tanzania, described elsewhere [11, 18]. All seven districts in Pwani region (intervention arm), and four districts from Morogoro and Lindi regions (comparison arm) were sampled. The comparison districts were selected to be comparable to intervention districts in terms of poverty and literacy rates, the rate of institutional deliveries, infant mortality, population per health facility, and the number of children under one year of age per capita [18]. Baseline data collection was done in January 2012, with a follow-up survey 13 months later.

### Sampling and data source

In the intervention arm, we included all 6 hospitals and 16 health centres that were eligible for the P4P scheme, and a random sample of 53 eligible dispensaries. A similar number of facilities were included in the comparison arm. Facilities were randomly sampled amongst those where P4P was implemented and matching comparison facilities were selected based on facility level of care, ownership, staffing levels, and case load [18]. To assess maternal and child health service utilisation in the population, we randomly sampled 20 households of women from the catchment area of each health facility who had delivered in the 12 months prior to the survey. In total, we surveyed 3000 households with eligible women in both arms at baseline, and a similar number in the follow-up survey. The household survey also collected information on maternal background characteristics (e.g. age, marital status, education occupation, religion, and number of births), and household characteristics (e.g. household size, health insurance status, and ownership of assets and housing particulars for assessing the household socioeconomic status).

### Outcome variables

Our outcome variables include the two incentivised services which we know from prior analysis improved significantly as a result of P4P: institutional deliveries and uptake of two doses of *intermittent preventive treatment* (IPT2) for malaria during antenatal care [11]. These were measured as binary outcomes for whether a woman gave birth in a health facility and received IPT2 during antenatal care, respectively.

### Generation of subgroups for distributional analyses

To examine the distribution of P4P effects on these two outcomes, we generated population subgroups based on individual and household-level characteristics, according to Andersen’s behavioural model of healthcare utilisation [3, 4]. In this study we only considered predisposing and enabling factors since data on perceived illness was not available. “Perceived illness” could also be argued to be of less relevance for maternal service utilisation outcomes, since study participants were largely healthy.

Subgroups of predisposing factors include: marital status (married vs. none), maternal age (15–49) years (below vs. above the median age of 25), education (no education vs. primary level/above), occupation (farmer vs. non-farmer), religion (Muslim vs. non-Muslim), number of births/parity (parity 1 vs. parity 2/above), and household size (below vs. above the median size of 5 members). Subgroups of enabling factors include: health insurance status (any insurance vs. none), place of residence (rural vs. urban district), and household wealth status subgroups. The wealth subgroups were generated from wealth scores derived by the principal component analysis based on 42 items of household characteristics and asset ownership (Appendix 1: Table 5) [29, 83]. The household wealth scores were generated separately for baseline and follow-up samples, since participants differed over time. Households were ranked by wealth scores from poorest (low score) to least poor and classified into three-equal sized groups (terciles): poorest, middle and least poor. Subgrouping based on five-equal sized groups (quintiles) were also generated to examine the sensitivity of the findings to different wealth subgroupings.

### Statistical analysis

We first compared the sample means of individual and household-level characteristics at baseline between intervention and comparison arms, and assessed whether the differences between arms were statistically significant by using t-tests. We then assessed the distribution of service utilisation outcomes at baseline across population subgroups by estimating the utilisation gap (i.e. a difference in average service use between two subgroups) [87]. We used t-tests to test whether the utilisation gaps were significantly different from zero.

To examine whether the effects of P4P on outcomes differed across population subgroups, we first performed subgroup analyses to identify the P4P effect on each subgroup, and then tested the significance of differential effects between subgroups through analysing the interaction effect. We identified the average effect of P4P on service utilisation by using a linear difference-in-differences regression model. This model compares the changes in outcomes over time between participants in the intervention and comparison arms as specified in Eq. (1):

1 $$ {Y}_{ijt}={\beta}_0+{\beta}_1\left(P4{P}_j\times {\delta}_t\right)+{\beta}_2{\delta}_t+{\beta}_3{X}_{ijt}+{\gamma}_i+{\varepsilon}_{ijt} $$

where *Y*_*ijt*_ is the utilisation outcome (institutional deliveries or uptake of IPT2) of individual *i* in facility *j’s* catchment area and at time *t.* The intervention dummy variable *P*4*P*_*j*_ takes the value 1 if a facility is in the intervention arm and 0 if it is in the comparison arm. The unobserved time invariant facility characteristics *γ*_*j*_ were controlled for through facility fixed-effects estimation; and included *δ*_*t*_ for year fixed effects. We also controlled for individual and household-level covariates *X*_*ijt*_ (age, education, occupation, religion, marital status, parity, insurance status, household size, and household wealth status) as potential confounders. The error term is *ε*_*ijt*_. We clustered the standard errors at the facility level, or facility catchment area, to account for serial correlation of *ε*_*ijt*_ at the facility level. The effect of P4P on utilisation for each subgroup is given by *β*_1_.

To test the significance of an eventual differential effect across subgroups, we included a three-way interaction term between the average treatment effect (*P*4*P*_*j*_ × *δ*_*t*_) and a subgrouping variable *G*_*i*_ (based on predisposing and enabling factors). The associated two-order interaction terms were also included in the model. The coefficient of interest is *β*_4_ which indicates the differential effect of P4P across subgroups as shown in Eq. (2):

2 $$ {Y}_{ijt}={\beta}_0+{\beta}_1\left(P4{P}_j\times {\delta}_t\right)+{\beta}_2{\delta}_t+{\beta}_3{X}_{ijt}+{\beta}_4\left(P4{P}_j\times {\delta}_t\times {G}_{ijt}\ \right)+{\beta}_5\left(P4{P}_j\times {G}_{ijt}\right)+{\beta}_6\left({G}_{ijt}\times {\delta}_t\right)+{\gamma}_j+{\varepsilon}_{ijt} $$

The use of the difference-in-difference approach to estimate the effect of P4P on outcomes relies on the key identifying assumption that the trends in outcomes would be parallel across study arms in the absence of the intervention [41]. While this can never be formally tested, we supported the assumption by verifying that the pre-intervention trends in utilisation outcomes at the household level were parallel across study arms as described elsewhere [11]. By surveying women who had delivered in the past 12 months at baseline, four longitudinal outcomes were generated and used to verify the assumption: share of institutional deliveries, caesarean section deliveries, women who breastfeed within one hour of birth, and women who paid for delivery care.

We further performed several robustness checks. First, we re-estimated the P4P differential effect by using wealth quintiles instead of wealth terciles to examine whether the results were sensitivity to wealth group classification. We also generated wealth status subgroups for each study arm and re-estimated the P4P differential effect by arm-based wealth subgroups to avoid the pre-existing baseline imbalance in wealth status between arms. Second, we re-estimated the regression model by including three-way interactions with categorical variable which gives multiple subgroups (e.g. education levels, occupation categories, parity groups and age groups) instead of interactions with binary variables (e.g. married vs. none). Third, we applied a non-linear logit model instead of linear model because of binary outcome variables. Fourth, we clustered the standard errors at the district level instead of facility level and used a bootstrapping method to adjust for the small number of clusters [20]. All the analyses were performed by using STATA version 13.

## Results

The majority of individual and household characteristics were similar across intervention and comparison arms at baseline (Table 2). Exceptions were women in the intervention arm, who were more likely to be married, non-farmers, and Muslim; and their households were more likely to be poor than their counterparts in the comparison arm.

**Table 2:** Baseline individual woman and household characteristics by study arms

Characteristics	Description/subgroup	Intervention arm (*n* = 1376)	Comparison arm (*n* = 1468)	Difference
Panel A: Predisposing factors				
Marital status	=1 for married woman (%)	69.9	64.2	5.7^b^
Age	Mean maternal age (15–49) years [SD]	26.5 [6.7]	26.3 [6.5]	0.2
Age	=1 for younger below median age (25 years) (%)	50.9	50.5	0.4
Education	=1 for primary education/above (%)	80.3	80.2	0.1
Occupation	=1 for farming activities (%)	46.0	54.5	–8.5^b^
Religion	=1 for Muslim woman (%)	86.5	66.6	19.9^a^
Parity	Mean number of births [SD]	2.7 [1.8]	2.6 [1.7]	0.1
Parity	=1 for one birth (%)	32.4	31.6	0.8
Household size	Mean number of household members [SD]	4.7 [1.8]	4.8 [1.8]	−0.1
Household size	=1 for small/below the median size of 5 members (%)	51.1	50.5	0.6
Panel B: Enabling factors				
Health insurance status	=1 for insured woman (%)	8.6	8.5	0.1
Household wealth status	Mean household wealth index [SD]	−0.43 [2.7]	0.34 [3.3]	−0.77^b^
Wealth status –tercile 1	=1 for poorest household (%)	38.3	29.4	8.9^b^
Wealth status –tercile 2	=1 for middle wealth household (%)	33.6	33.3	0.3
Wealth status –tercile 3	=1 for least poor household (%)	28.1	37.3	−9.2^b^
Place of residence	=1 for rural district (%)	79.3	84.1	−4.8

*SD*=Standard Deviation; Subgroups of predisposing factors include: marital status (married vs. none), maternal age (15–49) years (below vs. above the median age of 25), education (no education vs. primary level/above), occupation (farmer vs. non-farmer), religion (Muslim vs. non-Muslim), number of births/parity (parity 1 vs. parity 2/above), and household size (below vs. above the median size of 5 members); Subgroups of enabling factors include: health insurance status (any insurance vs. none), place of residence (rural vs. urban district), and household wealth status subgroups (wealth terciles); ^a^denotes significance at 1%, ^b^at 5%, and ^c^at 10% level

The baseline rates of institutional deliveries in both arms were significantly lower for women in the poorest and middle wealth households, and for women who were illiterate, farmers, with parity greater than one than for their counterpart women (Table 3). The rate of institutional deliveries was also higher among intervention women with health insurance and from smaller households, as well as among urban women in the comparison arm than among their counterparts. The baseline uptake of IPT2 was generally similar across arms and population subgroups, except married women in the comparison arm, who were more likely to receive IPT2 than unmarried women (Table 3).

**Table 3:** Baseline levels of service utilisation by subgroups across study arms

Outcome variable/ subgrouping variable	Intervention arm			Comparison arm		
	Yes	No	Gap	Yes	No	Gap
	(1)	(2)	(3)	(4)	(5)	(6)
OUTCOME 1: Institutional deliveries	(n = 1376)			(n = 1468)		
*Predisposing factors*						
Married woman (%)	84.8	84.7	0.1	86.7	87.0	−0.3
Woman below median age of 25 years/younger (%)	85.4	84.2	1.2	87.3	86.4	0.9
Woman with primary education/above (%)	85.9	80.4	5.5^b^	89.8	74.8	15.0^a^
Woman doing farming for occupation (%)	79.1	89.6	−10.5^a^	82.6	91.9	−9.3^a^
Muslim woman (%)	84.7	85.4	−0.7	87.5	85.5	2.0
Woman with one birth/parity 1 (%)	90.1	82.3	7.8^a^	92.5	84.3	8.2^a^
Household size below the median size of 5 members (%)	87.2	82.3	4.9^b^	87.3	86.4	0.9
*Enabling factors*						
Woman with any health insurance (%)	89.9	84.3	5.6^c^	83.3	87.1	−3.8
Household with poorest wealth status (Tercile 1) (%)	83.3	91.7	−8.4^a^	80.5	94.2	−13.7^a^
Household with middle wealth status (Tercile 2) (%)	80.8	91.7	−10.9^a^	84.2	94.2	−10.0^a^
Household in rural district (%)	83.9	88.0	−4.1	85.8	92.3	−6.5^c^
OUTCOME 2: Uptake of IPT2	(*n* = 1029)			(*n* = 1.199)		
*Predisposing factors*						
Married woman (%)	51.0	47.0	4.0	59.3	51.7	7.6^b^
Woman below median age of 25 years/younger (%)	48.7	51.1	−2.4	55.5	57.6	−2.1
Woman with primary education/above (%)	50.9	45.1	5.8	57.5	52.9	4.6
Woman doing farming for occupation (%)	48.5	51.1	−2.6	56.3	56.9	−0.6
Muslim woman (%)	49.9	50.4	−0.5	58.2	53.5	4.7
Woman with one birth/parity 1 (%)	48.0	50.8	−2.8	57.9	56.1	1.8
Household size below the median size of 5 members (%)	50.7	49.1	1.6	55.3	57.9	−2.6
*Enabling factors*						
Woman with any health insurance (%)	45.6	50.4	−4.8	61.6	56.1	5.5
Household with poorest wealth status (Tercile 1) (%)	47.8	49.6	−1.8	59.7	54.2	5.5
Household with middle wealth status (Tercile 2) (%)	52.6	49.6	3.0	56.9	54.2	2.7
Household in rural district (%)	50.4	48.1	2.3	56.7	56.4	0.3

We used a t-test to test the null hypothesis of a gap (column 3 and 6) equals to zero; Tercile 3 (least poor) was the reference category for Tercile 1 and 2; ^a^denotes significance at 1%, ^b^at 5%, and ^c^at 10% level

P4P significantly increased the rate of institutional deliveries among women in the poorest and in the middle wealth status households, but not among women in the least poor households (Table 4). However, when compared with the least poor subgroup, the effect of P4P was only marginally greater among women in the middle wealth status households only (*p* = 0.094 for differential effect) (Table 4). The effect of P4P on institutional deliveries was also significantly higher among women in rural districts compared to women in urban districts (*p* = 0.028 for differential effect), and among uninsured than insured women (*p* = 0.001 for differential effect). There were no differential effects of P4P on institutional deliveries among other subgroups, and no differential effects of P4P on the IPT2 outcome across any population subgroups (Table 4).

**Table 4:** Effect of P4P on service utilisation outcomes by population subgroups

Subgrouping variables	Institutional deliveries			Uptake of IPT2		
	Average subgroup effect		Differential effect test (*p*-value)	Average subgroup effect		Differential effect test (*p*-value)
	N	Beta		N	Beta	
Marital status						
Married	3869	7.7^a^	(*p* = 0.564)	3253	10.2^a^	(*p* = 0.927)
Unmarried	1878	9.1^b^		1504	9.1	
Maternal age						
Younger below the median age	2914	8.5^a^	(*p* = 0.553)	2336	9.6^b^	(*p* = 0.841)
Older above the median age	2833	7.2^b^		2421	9.8^b^	
Education						
Some education	4611	8.9^a^	(*p* = 0.378)	3877	9.3^a^	(*p* = 0.780)
No education/illiterate	1136	5.9		880	16.5^c^	
Occupation						
Farmer	2950	11.5^a^	(*p* = 0.133)	2434	16.0^a^	(*p* = 0.167)
Non-farmer	2797	5.6^b^		2323	5.6	
Religion						
Muslim	4376	9.7^a^	(*p* = 0.435)	3623	10.5^a^	(*p* = 0.562)
Non-Muslim	1371	3.9		1134	6.0	
Parity/births						
One birth	1886	9.7^a^	(*p* = 0.517)	1510	9.3^c^	(*p* = 0.882)
Two or more births	3861	7.6^a^		3247	10.3^a^	
Household size by members						
Small size (< 5)	2996	5.1^c^	(*p* = 0.173)	2476	7.7^c^	(*p* = 0.964)
Large size (≥5)	2751	10.4^a^		2281	9.9^b^	
Health insurance						
Insured	475	−7.6	(p = 0.001)	429	20.1^c^	(*p* = 0.932)
Uninsured	5272	9.7^a^		4328	10.4^a^	
Household wealth subgroups						
Tercile 1 (poorest)	1940	11.4^b^	(*p* = 0.232)	1559	14.5^b^	(*p* = 0.158)
Tercile 2 (middle)	1916	10.2^a^	(p = 0.094)	1576	16.2^a^	(*p* = 0.149)
Tercile 3 (least poor)	1891	3.7	Reference	1622	2.6	Reference
Place of residence						
Rural district	4694	9.9^a^	(p = 0.028)	3851	11.4^a^	(*p* = 0.349)
Urban district	1053	0.9		906	3.3	

Beta is the estimated P4P effect on a specific subgroup in percentage point after controlling for a year dummy, facility-fixed effects, and individual and household-level covariates (age, education, occupation, religion, marital status, parity, health insurance status, household size, and household wealth status); Each cell for Beta and differential effect reports the result from a separate regression; Differential effect test is a t-test of the null that the coefficient on the three-way interaction between the P4P effect and subgrouping indicator is zero; ^a^denotes significance at 1%, ^b^at 5%, and ^c^at 10% level

Our results were generally consistent following robustness checks. When we used wealth quintiles instead of terciles, the effect of P4P on deliveries was significantly higher in lower quintiles (indication of pro-poor) compared to the effect in the top quintile (least poor), but the results on IPT2 remained the same (Appendix 2: Table 6). When we used the arm-based wealth subgroups, the differential effect by quintiles on both outcomes remained broadly unchanged, but the differential effect by terciles on deliveries disappeared and appeared marginally for IPT2 (Appendix 2: Table 6). The effect of P4P on both outcomes remained equally distributed across categorical subgroups of education, occupation, parity and age (Appendix 3: Table 7). Some changes in the results were noted with the use of a logit model, the pro-middle wealth and pro-rural effect on deliveries disappeared but all other results including the pro-uninsured effect remained the same (Appendix 4: Table 8). When standard errors were clustered at the district-level instead of at facility-level, the differential effect on deliveries by health insurance and wealth status disappeared, and women from larger households increased institutional deliveries more than their counterparts, but all other results including the pro-rural effect remained unchanged (Appendix 5: Table 9).

## Discussion

This study examined the distribution of P4P effects on service utilisation outcomes across population subgroups in Tanzania. This is the first study in LMICs to examine who is really benefiting from the effects of P4P across a broad range of population characteristics which aligns with the social determinants of health framework. We found that P4P increased institutional deliveries more among women in middle wealth status households, among the uninsured, and among women living in rural areas than among wealthier, insured, and urban residing women. However, these differential effects were sensitive to the analytical specifications used during the robustness checks. The effect of P4P on IPT2 was equally distributed across population subgroups, and was robust across various analytical specifications. Our results show a declining trend in inequality to access institutional deliveries since service use improved most for subgroups which initially showed low utilisation rates; while the absence of inequality in uptake of IPT2 at baseline maintained after the introduction of P4P.

The greater impact of P4P on the use of institutional deliveries among women in the middle wealth households and uninsured than wealthier and insured respectively, is likely in part due to the increased adherence to user fee exemption policy among public facilities as well as the improved availability of drugs, minimising the need to pay for drugs in private pharmacies [5, 10, 11, 27, 39, 43, 45, 85, 86, 90]. The worse-off groups which experienced a greater P4P effect were also more responsive to a change in healthcare costs [33, 49]. This is consistent with our conceptual framework and demand theory, whereby the supply-side responses of reducing the financial barriers to access delivery care in turn stimulated the demand-side responses on service utilisation mostly among the disadvantaged population.

The finding that the increased uptake of IPT2 was similar across population subgroups may be explained by the already almost universal access to one antenatal care visit in Tanzania (above 97%) [11, 75, 76]. In an effort to achieve the IPT2 target, providers likely encouraged women to return for subsequent antenatal care visits to receive at least two doses of IPT. This represents a relatively easy task for most providers because continuation of care needs less effort than its initiation [34]. Although the provision of IPT is within the control of providers, it also depends on the available stock of antimalarial drugs for IPT. Another reason for the lack of differential effect on IPT2 may have been the pre-existing balance in the uptake of IPT2 across population subgroups at baseline. This is the first study to examine whether P4P had a differential effect on the uptake of IPT for malaria during antenatal care in LMICs. In Burundi, Bonfrer et al. [17] examined the differential effect of P4P on other contents of antenatal care and found a pro-rich effect on blood pressure measurement and a lack of differential effect on the uptake of anti-tetanus vaccination across socioeconomic groups.

The pro-middle wealth effect of P4P on institutional deliveries, as an indication of being pro-poor, is contrary to the pro-rich effect on deliveries reported in Burundi [17], Rwanda [46] and Cambodia [77]. The pro-rich effect in Cambodia was attributed to the lack of effective demand among the poorest women due to user fees [77]; whereas in Burundi it was attributed to other costs like transport because the user fees for deliveries were removed prior to P4P [17]. However, a pilot study in Burundi [16] and a study using demographic and health survey (DHS) data in Rwanda [62] found no differential effect on deliveries by household wealth status; and the results in the later study were attributed to low and uniform coverage of services at baseline. In the Democratic Republic of Congo providers implementing P4P negotiated user fees with communities and raised revenues without hurting the poorest [73], but the equity effects of this approach were not assessed empirically. Further evidence of a pro-poor effect of P4P has been shown on immunisation services in Burundi [17], and on quality of care improvement in high-income countries especially in the United Kingdom [2, 14, 23, 24, 80].

Moreover, our study found that institutional deliveries improved more in rural than in urban areas, while there was no differential effect on institutional deliveries by place of residence in Rwanda [62]. In Rwanda, the minimal number of urban clusters compared to rural clusters were thought to limit the power to detect the differential effect by place of residence [62], while our study had a slightly higher number of urban clusters compared to Rwanda (i.e. 28 versus 22 urban clusters). In the United Kingdom, the effect of P4P on quality of care was greater in urban areas than in rural areas [36, 42], while there was no differential effect of P4P on quality of care by rural–urban area in the United States [67].

We found a greater P4P effect on institutional deliveries among uninsured women, whereas a greater effect on deliveries was found among women with health insurance in Rwanda [46] and a maternity care voucher in Cambodia [77]. The findings from Rwanda and Cambodia were attributed to reduced financial barriers to access care [46, 77], and this could be the case with a stronger enforcement of fee exemptions in Tanzania [11].

However, another study in Rwanda based on DHS, as nationally representative data, found no differential effect on deliveries by health insurance status [62]. A greater P4P effect on deliveries among uninsured women in Tanzania, is partly because the baseline institutional delivery rate was already higher among insured than uninsured women in the intervention arm. A further reason could be that uninsured women were more responsive to reduced healthcare costs compared to insured women who were already covered. It is also likely that the statistical power to detect the effect among women with insurance was limited because few women are insured in Tanzania [58], compared to other countries like Rwanda [50, 70].

Furthermore, we found a similar distribution of institutional delivery rates and IPT2 uptakes across age groups prior to P4P, and the effect of P4P was equally distributed across age groups, which is contrary to P4P studies in high-income countries as they found inequalities in quality of care across age groups existed and persisted after the introduction of P4P [2, 14, 24, 80].

Overall our findings imply that when P4P results in supply side responses that reduce demand-side barriers to accessing care, it can enhance equity in service utilisation. P4P also appears less likely to show a differential effect when there is a similar level of service utilisation in a given indicator across population subgroups prior to an intervention. This study supports the argument that P4P can enhance equity in access for services where there is a pre-existing inequity in coverage, and where efforts to remove the demand-side financial barriers to access care have been made [28, 31, 44, 57, 86]. Thus, to ensure P4P reduces inequities in access to care, policy makers should consider introducing complementary measures to reduce demand-side access barriers. P4P is likely to be most effective at reducing inequities in settings where they offer free health services or there is high coverage of pre-payment schemes.

To make progress towards universal health coverage and achieve sustainable development goal three especially in LMICs, more efforts are needed to stimulate demand for and supply of healthcare services [57, 86, 90]. Further insights on how supply and demand side interventions interact and complement each other to affect outcomes are needed. Moreover, because the social determinants of health as sources of inequalities emerge from different sectors, strategies within the health sector alone cannot reduce inequalities in access and use of health services [21, 54].

This study has a number of limitations. First, our study may have been underpowered to detect the effect of P4P in some groups, for example among insured women and urban residents, possibly due to the more limited sample size within sub groups. Second, our results of differential effects on deliveries by wealth status, health insurance and place of residence, were not consistent across all analytical specifications used in robustness checks (i.e. non-linear model, and district level clustering of standard errors). However, the differential effects on deliveries for other subgroups of social determinants, and differential effects on IPT2, were robust to all analytical specifications used. Third, our finding that P4P reduces inequalities in service utilisation might be reflective of a regression to the mean principle (a random fluctuation rather than a true causal effect) because of having a short term evaluation [7]. Lastly, we restricted our distributional analysis to the outcomes which improved significantly under P4P. Although the inequalities in service use may happen with an outcome which showed insignificant P4P effect on average, our focus was limited to how the increased average utilisation effects were distributed across population subgroups.

## Conclusion

In Tanzania, the effect of P4P on institutional deliveries was greater among women in middle wealth households, in rural areas and among the uninsured women than their counterparts. P4P effect on the uptake of IPT2 was equally distributed across population subgroups. Our finding suggests that P4P can enhance equitable healthcare access and use especially when the financial barriers to access care are reduced or removed.

## Appendix

### Appendix 1

**Table 5:** Items used to construct household wealth status score

**No.**	**Variable description**
1.	Asset: electricity
2.	Asset: working radio
3.	Asset: working television (TV)
4.	Asset: working DVD
5.	Asset: working mobile phone
6.	Asset: working landline phone
7.	Asset: working iron
8.	Asset: working refrigerator
9.	Asset: working wall watch
10.	Asset: sewing machine
11.	Asset: table
12.	Asset: sofa coach
13.	Asset: cupboard
14.	Asset: motorcycle
15.	Asset: car
16.	Household member with a bank account
17.	Number of sleeping rooms
18.	Source of drinking water: piped water
19.	Source of drinking water: borehole/ covered well
20.	Source of drinking water: open well
21.	Source of drinking water: spring water
22.	Source of drinking water: river/ dam/pond/lake
23.	Toilet type: flush toilet
24.	Toilet type: pit latrine
25.	Toilet type: no/ other toilet
26.	Source of cooking energy: electricity
27.	Source of cooking energy: kerosene/paraffin
28.	Source of cooking energy: charcoal
29.	Source of cooking energy: firewood
30.	Source of light: electricity
31.	Source of light: solar
32.	Source of light: kerosene/ paraffin
33.	Source of light: candle/ firewood
34.	Source of light: torch or other source
35.	Floor material: sand/earth/dung
36.	Floor material: cement
37.	Floor material: other
38.	Wall material: grass/poles/mud wall
39.	Wall material: bamboo with mud wall
40.	Wall material: sundried/ burnt bricks
41.	Wall material: cement blocks
42.	Wall material: stones with mud

### Appendix 2

**Table 6:** Effect of P4P on service utilisation by different categories of wealth status and by arm-based wealth subgroups

Wealth subgrouping variables	Institutional deliveries			Uptake of IPT2		
	Average subgroup effect		Differential effect test (p-value)	Average subgroup effect		Differential effect test (p-value)
	N	Beta		N	Beta	
*Panel A: Wealth subgroups*						
Three wealth subgroups (Terciles)						
T1	1940	11.4^b^	(p = 0.232)	1559	14.5^b^	(p = 0.158)
T2	1916	10.2^a^	(p = 0.094)^c^	1576	16.2^a^	(p = 0.149)
T3	1891	3.7	Reference	1622	2.6	Reference
Five wealth subgroups (Quintiles)						
Q1	1170	13.8^b^	(*p* = 0.079)^c^	929	13.6^c^	(*p* = 0.166)
Q2	1158	8.8^c^	(*p* = 0.069)^c^	939	16.3^b^	(*p* = 0.102)
Q3	1143	8.2^c^	(*p* = 0.034)^b^	938	21.8^a^	(*p* = 0.120)
Q4	1146	11.4^a^	(*p* = 0.015)^b^	979	14.4^b^	(*p* = 0.175)
Q5	1130	−0.5	Reference	972	1.9	Reference
*Panel B: Arm-based wealth subgroups*						
Three wealth subgroups (Terciles)						
AT1	1917	10.2^b^	(*p* = 0.293)	1540	13.8^b^	(*p* = 0.117)
AT2	1913	9.2^b^	(*p* = 0.156)	1568	18.3^a^	(*p* = 0.084)^c^
AT3	1917	3.9^c^	Reference	1649	2.5	Reference
Five wealth subgroups (Quintiles)						
AQ1	1149	15.3^a^	(*p* = 0.089)^c^	914	16.8^b^	(*p* = 0.108)
AQ2	1151	6.6	(*p* = 0.230)	935	15.2^b^	(*p* = 0.139)
AQ3	1147	12.3^b^	(p = 0.001)^a^	949	14.6^b^	(p = 0.156)
AQ4	1152	9.9^b^	(*p* = 0.022)^b^	972	7.7	(*p* = 0.310)
AQ5	1148	0.3	Reference	987	0.5	Reference

^a^denotes significance at 1%, ^b^at 5%, and ^c^at 10% level; Beta is the estimated P4P effect on a specific subgroup in percentage point after controlling for a year dummy, facility-fixed effects, and individual and household-level covariates (age, education, occupation, religion, marital status, parity, health insurance status, household size, and household wealth status); Each cell for Beta and differential effect reports the result from a separate regression; Differential effect test is a t-test of the null that the coefficient on the three-way interaction between the P4P effect and subgrouping indicator is zero

### Appendix 3

**Table 7:** Effect of P4P on service utilisation by subgroups for categorical variables

Subgrouping variables	Institutional deliveries			Uptake of IPT2		
	Average subgroup effect		Differential effect test (p-value)	Average subgroup effect		Differential effect test (p-value)
	N	Beta		N	Beta	
Education subgroups						
No education	1136	5.9	Reference	880	17.0^b^	Reference
Some primary	459	4.1	(*p* = 0.550)	355	9.1	(*p* = 0.479)
Primary/some secondary	3729	11.3^a^	(*p* = 0.157)	3148	12.1^a^	(*p* = 0.965)
Secondary/above	423	3.8	(*p* = 0.276)	374	−9.8	(*p* = 0.144)
Occupation subgroups						
Formal sector	113	−17.4	(*p* = 0.715)	99	−5.1	(*p* = 0.329)
Farmers	2950	11.6^a^	(*p* = 0.162)	2434	15.9^a^	(*p* = 0.777)
Self-employed	1167	7.7^b^	(*p* = 0.650)	996	1.1	(*p* = 0.132)
Unemployed	1517	3.9	Reference	1228	16.8^a^	Reference
Birth parity subgroups						
Parity 1	1886	9.8^a^	Reference	1510	9.3^c^	Reference
Parity 2	1353	3.4	(*p* = 0.215)	1123	7.0	(*p* = 0.583)
Parity 3	1029	10.9^b^	(*p* = 0.766)	868	0.4	(*p* = 0.317)
Parity 4	664	3.3	(*p* = 0.342)	570	3.2	(*p* = 0.567)
Parity 5+	815	13.3^c^	(*p* = 0.700)	686	30.0^a^	(*p* = 0.038)
Age subgroups						
Age (15–19) years	965	11.5^a^	Reference	726	19.2^b^	Reference
Age (20–24) years	1613	9.7^a^	(*p* = 0.366)	1322	4.2	(*p* = 0.708)
Age (25–29) years	1459	4.2	(*p* = 0.568)	1232	7.3	(*p* = 0.820)
Age (30–34) years	978	4.9	(*p* = 0.510)	846	10.3	(*p* = 0.666)
Age (35+) years	732	15.5^a^	(*p* = 0.446)	631	20.4^b^	(*p* = 0.218)

^a^denotes significance at 1%, ^b^at 5%, and ^c^at 10% level; Beta is the estimated P4P effect on a specific subgroup in percentage point after controlling for a year dummy, facility-fixed effects, and individual and household-level covariates (age, education, occupation, religion, marital status, parity, health insurance status, household size, and household wealth status); Each cell for Beta and differential effect reports the result from a separate regression; Differential effect test is a t-test of the null that the coefficient on the three-way interaction between the P4P effect and subgrouping indicator is zero

### Appendix4

**Table 8:** Effect of P4P on service utilisation by subgroups –using the non–linear logit model

**Subgrouping variables**	Institutional deliveries			Uptake of IPT2		
	Average subgroup effect		Differential effect test (p-value)	Average subgroup effect		Differential effect test (p-value)
	N	(dy/dx)		N	(dy/dx)	
Marital status						
Married	3385	9.2^a^	(*p* = 0.503)	3253	9.2^a^	(*p* = 0.935)
Unmarried	1338	13.3^a^		1481	9.8^c^	
Maternal age						
Younger below the median age	2361	11.2^a^	(*p* = 0.492)	2336	9.2^b^	(*p* = 0.830)
Older above the median age	2325	9.1^a^		2421	9.5^b^	
Education						
Some education	4021	10.9^a^	(*p* = 0.070)	3877	8.6^a^	(*p* = 0.793)
No education/illiterate	900	9.1		816	16.5^c^	
Occupation						
Farmer	2638	13.4^a^	(*p* = 0.590)	2396	16.0^a^	(p = 0.149)
Non-farmer	2126	7.5^b^		2295	5.3	
Religion						
Muslim	3991	10.8^a^	(*p* = 0.497)	3614	9.7^a^	(*p* = 0.554)
Non-Muslim	980	5.6		1061	7.8	
Parity/births						
One birth	1180	15.2^a^	(*p* = 0.122)	1476	9.9^c^	(*p* = 0.939)
Two or more births	3436	9.3^a^		3247	10.0^a^	
Household size by members						
Small size (< 5)	2381	7.3^b^	(*p* = 0.320)	2464	7.6^c^	(*p* = 0.903)
Large size (≥5)	2299	12.8^a^		2281	9.1^b^	
Health insurance						
Insured	171	−20.7	(*p* = 0.012)	315	18.3	(*p* = 0.900)
Uninsured	4820	11.1^a^		4328	10.1^a^	
Household wealth status						
Tercile 1 (poorest)	1656	13.4^b^	(*p* = 0.894)	1508	13.2^b^	(*p* = 0.145)
Tercile 2 (middle)	1528	12.7^a^	(*p* = 0.737)	1539	17.1^a^	(*p* = 0.106)
Tercile 3 (least poor)	1066	8.2^b^	Reference	1599	2.4	Reference
Place of residence						
Rural district	4387	11.3^a^	(*p* = 0.152)	3851	11.2^a^	(*p* = 0.268)
Urban district	787	1.6		906	1.7	

Non-linear logit model with FE, covariates, clustering at HF level; Logit with FE cuts down the sample size; dy/dx is the estimated partial P4P effect on a specific subgroup in terms of marginal effect after controlling for a year dummy, facility-fixed effects, and individual and household-level covariates (age, education, occupation, religion, marital status, parity, health insurance status, household size, and household wealth status); Each cell for dy/dx and differential effect reports the result from a separate regression; Differential effect test is a t-test of the null that the coefficient on the three-way interaction between the P4P effect and subgrouping indicator is zero; ^a^ denotes significance at 1%, ^b^at 5%, and ^c^ at 10% level

### Appendix 5

**Table 9:** Effect of P4P on service utilisation by subgroups –using district-level clustering of Standard Errors

	Institutional deliveries			Uptake of IPT2		
**Subgrouping variables**	Average subgroup effect		Differential effect test (p-value)	Average subgroup effect		Differential effect test (p-value)
	N	Beta		N	Beta	
Marital status						
Married	3869	7.7	(*p* = 0.580)	3253	10.2^a^	(*p* = 0.960)
Unmarried	1878	9.1		1504	9.1	
Maternal age						
Younger below the median age	2914	8.5	(*p* = 0.565)	2336	9.6^b^	(*p* = 0.790)
Older above the median age	2833	7.2^b^		2421	9.8	
Education						
Some education	4611	8.9^c^	(*p* = 0.400)	3877	9.3^a^	(*p* = 0.800)
No education/illiterate	1136	5.9		880	16.5	
Occupation						
Farmer	2950	11.5^c^	(*p* = 0.140)	2434	16.0^a^	(*p* = 0.060)
Non-farmer	2797	5.6		2323	5.6	
Religion						
Muslim	4376	9.7^a^	(*p* = 0.600)	3623	10.5^b^	(*p* = 0.440)
Non-Muslim	1371	3.9		1134	6.0	
Parity/births						
One birth	1886	9.7^b^	(*p* = 0.455)	1510	9.3^a^	(*p* = 0.895)
Two or more births	3861	7.6^c^		3247	10.3	
Household size by members						
Small size (< 5)	2996	5.1	(*p* = 0.045)	2476	7.7^c^	(*p* = 0.925)
Large size (≥5)	2751	10.4^c^		2281	9.9^a^	
Health insurance						
Insured	475	−7.6	(*p* = 0.225)	429	20.1^a^	(p = 0.965)
Uninsured	5272	9.7^b^		4328	10.4^a^	
Household wealth status						
Tercile 1 (poorest)	1940	11.4	(p = 0.400)	1559	14.5^a^	(p = 0.120)
Tercile 2 (middle)	1916	10.2^c^	(*p* = 0.125)	1576	16.2^a^	(*p* = 0.050)
Tercile 3 (least poor)	1891	3.7	Reference	1622	2.6	Reference
Place of residence						
Rural district	4694	9.9^c^	(*p* = 0.080)	3851	11.4^b^	(*p* = 0.430)
Urban district	1053	0.9		906	3.3	

## Abbreviations

DHS: Demographic Health Survey
IPT: Intermittent preventive treatment
LMICs: Low- and middle-income countries
MCH: Maternal and child health
MoHSW: Ministry of Health and Social Welfare
P4P: Payment for performance
USD: United States dollar
